# Age-related associations of hypertension and diabetes mellitus with chronic kidney disease

**DOI:** 10.1186/1471-2369-10-17

**Published:** 2009-06-30

**Authors:** Tareq M Islam, Caroline S Fox, Devin Mann, Paul Muntner

**Affiliations:** 1 Department of Epidemiology, Tulane University School of Public Health and Tropical Medicine, New Orleans, Louisiana, USA; 2 National Heart, Lung, and Blood Institute's Framingham Heart Study, Framingham, Massachusetts, USA; 3 Division of Endocrinology, Metabolism, and Diabetes, Department of Medicine, Harvard Medical School, Boston, Massachusetts, USA; 4 Department of Medicine, Mount Sinai School of Medicine, New York, USA; 5 Department of Epidemiology, University of Alabama at Birmingham, Birmingham, AL, USA

## Abstract

**Background:**

Studies suggest end-stage renal disease incidence and all-cause mortality rates among patients with chronic kidney disease (CKD) differ by age. The association of diabetes mellitus and hypertension with CKD across the adult lifespan is not well established.

**Methods:**

Data from NHANES 1999–2004 were used to determine the association of risk factors for stage 3 or 4 CKD (n = 12,518) and albuminuria (n = 12,778) by age grouping (20 to 49, 50 to 69, and ≥70 years). Stage 3 or 4 CKD was defined as an estimated glomerular filtration rate of 15 to 59 ml/min/1.73 m^2 ^and albuminuria as an albumin to creatinine ratio ≥30 mg/g.

**Results:**

For adults 20 to 49, 50 to 69 and ≥70 years of age, the prevalence ratios (95% confidence interval) of stage 3 or 4 CKD associated with hypertension were 1.94 (0.86 – 4.35), 1.51 (1.09 – 2.07), 1.31 (1.15 – 1.49), respectively (p-trend = 0.038). The analogous prevalence ratios (95% confidence interval) were 3.01 (1.35 – 6.74), 1.61 (1.15 – 2.25), 1.40 (1.15 – 1.69), respectively, for diagnosed diabetes mellitus (p-trend = 0.067); and 2.67 (0.53 – 13.4), 1.35 (0.69 – 2.63), 1.08 (0.78 – 1.51), respectively, for undiagnosed diabetes mellitus (p-trend = 0.369). The prevalence ratios of albuminuria associated with hypertension and diagnosed and undiagnosed diabetes mellitus were lower at older age (each p < 0.05).

**Conclusion:**

Among US adults, diabetes mellitus and hypertension are associated with CKD and albuminuria regardless of age. However, the associations were stronger at younger ages.

## Background

Chronic kidney disease (CKD), defined by the presence of albuminuria or reduced glomerular filtration rate (GFR), is common, and increasing, among adults in the United States and worldwide. [1-3] The incidence and prevalence of CKD increase markedly at older age.[2,4] For example, in the Framingham Heart Study, the risk of developing stage 3 CKD was 2.36 times higher for each 10 years older age.[4] Additionally, the prevalence of stage 1 or 2 and stage 3 or 4 CKD among US adults has been reported to be 3.3 and 54 times higher, respectively, for adults ≥70 versus 20 to 39 years of age.[2]

Although the incidence and prevalence of CKD increases with age, several reports have suggested that the relative risk of adverse outcomes including end-stage renal disease (ESRD) and mortality associated with CKD are attenuated at older age.[5,6] While these studies have provided important insight on outcomes among patients with established CKD, few data are available on the importance and differential impact of risk factors for CKD across the full adult lifespan. Similar to the age-related association of CKD with ESRD and mortality, the relationship between CKD and major risk factors may differ by age. To determine the association of risk factors for stage 3 or 4 CKD and albuminuria by age grouping, we analyzed data from the National Health and Nutrition Examination Survey (NHANES) 1999–2004. We hypothesized that hypertension and diabetes mellitus are important risk factors for CKD regardless of age but, similar to patterns observed for outcomes among adults with CKD, these associations would be stronger for younger adults.

## Methods

NHANES 1999–2004 is a cross-sectional nationally representative survey of the non-institutionalized civilian population of the United States.[7] Briefly, this study employs a multi-stage stratified probability sample based on selection of counties, blocks, households, and persons within households. Mexican-Americans, non-Hispanic blacks, and older adults were over-sampled in order to improve the precision of estimates for these groups. NHANES 1999–2004 consisted of an in-home interview and a medical evaluation and blood sample collection in a mobile examination center. Overall, 15,332 participants completed the medical evaluation and study interview. Participants without serum creatinine measurements, estimated GFR (eGFR) < 15 ml/min/1.73 m^2^, and pregnant women were excluded from the analysis of stage 3 or 4 CKD, resulting in a sample size of 12,518 participants. For the analysis of albuminuria, individuals missing urinary albumin or urinary creatinine measurements and pregnant or menstruating women were excluded, resulting in a total sample size of 12,778 participants. Women and non-Hispanic blacks were more likely than men and non-Hispanic whites to be missing serum creatinine while older participants and non-Hispanic blacks were more likely than younger individuals and non-Hispanic whites to be missing urinary albumin or urinary creatinine data.

Of relevance to the current analysis, variables collected during the in-home interview were age, race-ethnicity, gender, cigarette smoking, a previous diagnosis of diabetes mellitus, cardiovascular disease (myocardial infarction or stroke) and pharmacologic treatment for hypertension, high cholesterol, or diabetes mellitus. Self-report of a prior diagnosis of diabetes mellitus with current use of an oral hypoglycemic agent or insulin was used to define diagnosed diabetes mellitus. Body mass index (BMI) was calculated as weight in kilograms divided by height in meters squared; obesity was defined as BMI ≥30 kg/m^2^. Three blood pressure measurements were obtained using a standard protocol and hypertension was defined as an average systolic or diastolic blood pressure ≥140 mmHg or 90 mmHg, respectively, or current use of blood pressure lowering medication.[8]

Total cholesterol was measured with the Hitachi 704 Analyzer; high cholesterol was defined as total cholesterol ≥240 mg/dL or pharmacologic lipid lowering treatment. A sample of NHANES 1999–2004 participants were assigned to attend a morning study visit to the mobile examination center after an overnight fast. For participants who attended the morning study visit, glucose was measured on previously frozen plasma. Among participants without diagnosed diabetes mellitus who attended a morning study visit and fasted 8 hours or longer (N = 5597 for the stage 3 or 4 CKD analyses and N = 5507 for the albuminuria analyses), undiagnosed diabetes mellitus was defined as plasma glucose ≥126 mg/dL.

Serum creatinine was measured using the modified kinetic method of Jaffe (Hitachi 917 analyzer). Serum creatinine concentrations were calibrated to the assays used for the development of the Modification of Diet in Renal Disease (MDRD) study equation.[9] The simplified MDRD study equation was used to calculate eGFR.[10] Individuals with an eGFR of 15 to 59 ml/min/1.73 m^2 ^were considered to have stage 3 or 4 CKD.[11] Urinary albumin was measured using a solid-phase fluorescence immunoassay; urinary creatinine was measured using modified kinetic method of Jaffe (Beckman Coulter Synchron AS/Astra Analyzer). Albuminuria was defined as a urinary albumin to urinary creatinine ratio = 30 mg/g.[11] The results were markedly consistent when gender specific cut-points (urinary albumin to urinary creatinine ratio = 30 mg/g for women and = 20 mg/g for men) were used to define albuminuria and therefore are not presented.[12]

The protocols for NHANES 1999–2004 were approved by the National Center for Health Statistics of the Centers for Disease Control and Prevention Institutional Review Board. All participants provided written informed consent.

### Statistical Methods

The prevalence of stage 3 or 4 CKD and albuminuria, separately, was calculated by age grouping (20 to 49 years, 50 to 69 years, and ≥70 years) for the overall population and for individuals with and without a history of hypertension and diabetes mellitus, separately. Characteristics of the population were calculated, stratified by age grouping for participants with and without stage 3 or 4 CKD and with and without albuminuria, separately. The statistical significance of differences in the means and prevalence estimates for participants with and without stage 3 or 4 CKD, and with and without albuminuria, was determined using linear and logistic regression models, respectively, after adjustment for age, modeled as a continuous variable. Prevalence ratios of stage 3 or 4 CKD and albuminuria, separately, associated with non-Hispanic black race-ethnicity, female gender, cigarette smoking, obesity, hypertension, high cholesterol, diagnosed and undiagnosed diabetes mellitus, and a history of cardiovascular disease were calculated for each age grouping, separately, using log-binomial regression models. All regression models included age, race-ethnicity, gender, hypertension and diagnosed diabetes mellitus. Undiagnosed diabetes mellitus was not included in all regression models as fasting plasma glucose was measured only on a sub-sample of NHANES participants. The statistical significance of the linear trends across age group was assessed by including an interaction term (e.g., age group*hypertension) in the regression model. For these models, each person within an age grouping was assigned the median age for that grouping (i.e., 36, 57, and 76 years for participants 20 to 49, 50 to 69, and ≥70 years, respectively).

Sample weights that account for the complex survey design of NHANES including unequal probabilities of selection, over-sampling, and non-response were applied for all analyses using SUDAAN (Version 9.1; Research Triangle Institute, Research Triangle Park, NC). Standard errors were estimated using the Taylor series linearization method.

## Results

### Risk factors for stage 3–4 chronic kidney disease by age grouping

Stage 3 or 4 CKD was progressively more common at older age with a prevalence of 1.4%, 9.9% and 38.3% among adults 20 to 49, 50 to 69 and ≥70 years of age, respectively. The age-specific prevalence of stage 3 or 4 CKD at 20 to 49, 50 to 69 and ≥70 years of age was 1.5%, 11.7%, and 39.0%, respectively, among individuals with diagnosed or undiagnosed diabetes mellitus or hypertension and 1.0%, 6.6% and 27.8%, respectively, among individuals without diabetes mellitus or hypertension.

Table S1 presents demographic characteristics and the prevalence and level of risk factors among individuals with and without stage 3 or 4 CKD stratified by age group [Additional file 1]. Within each age grouping, the difference in the prevalence of cigarette smoking was not significantly different among participants with and without CKD. Among participants 50 to 69 and ≥70 years of age but not their counterparts 20 to 49 years, obesity, hypertension, high cholesterol, and a history of cardiovascular disease were more common among those with compared to without stage 3 or 4 CKD. Within each age group, the prevalence of diagnosed diabetes mellitus was higher among participants with stage 3 or 4 CKD. Although undiagnosed diabetes mellitus was more common among adults with stage 3 or 4 CKD within each age group, this was not statistically significant after age-adjustment.

No patterns were present across age groups in the prevalence ratio of stage 3 or 4 CKD associated with gender, race-ethnicity, cigarette smoking, obesity, or high cholesterol [Additional file 2]. After adjustment for age, race-ethnicity, gender, and diagnosed diabetes mellitus, the prevalence ratio of stage 3 or 4 CKD associated with hypertension was lower at older age (p-trend = 0.038). Similarly, after adjustment for age, race-ethnicity, gender, hypertension, and diagnosed diabetes mellitus, the prevalence ratio of stage 3 or 4 CKD was lower at older age for cardiovascular disease (p-trend = 0.018). Although not statistically significant, a trend towards lower prevalence ratios of stage 3 or 4 CKD at older age were present at older age for diagnosed (p-trend = 0.067) and undiagnosed diabetes mellitus (p-trend = 0.369).

### Risk factors for albuminuria by age grouping

Similar to stage 3 or 4 CKD, albuminuria was more common at older age (5.8%, 11.4% and 22.7% among adults 20 to 49, 50 to 69 and ≥70 years of age, respectively). Among individuals 20 to 49, 50 to 69 and ≥70 years of age, the prevalence of albuminuria was 14.0%, 14.9%, and 26.3%, respectively, among individuals with hypertension or diagnosed or undiagnosed diabetes mellitus and 3.7%, 6.9% and 14.0%, respectively, for those without diagnosed or undiagnosed diabetes mellitus or hypertension.

Although not statistically significant for all age groups, cigarette smoking and a history of cardiovascular disease were more common among participants with albuminuria within each age grouping [Additional file 3]. In contrast, among adults = 70 years of age, high cholesterol was less common among those with albuminuria. Among all age groups, the prevalence of obesity, hypertension, and diagnosed and undiagnosed diabetes mellitus was higher among those with, compared to without, albuminuria.

Although no patterns in the prevalence ratio of albuminuria associated with black race-ethnicity, cigarette smoking, and cardiovascular disease were present across age group, progressively weaker associations of albuminuria with female gender, obesity, and high cholesterol at older age groups were present [Additional file 4]. After adjustment for age, race-ethnicity, gender, and diagnosed diabetes mellitus, the prevalence ratio of albuminuria associated with hypertension was progressively lower in the older age groupings (p = 0.019). Similar patterns of lower prevalence ratios of albuminuria at older age groupings were present for diagnosed and undiagnosed diabetes mellitus (p < 0.001 and p = 0.003, respectively).

## Discussion

In the current study, associations were present between diagnosed diabetes mellitus and stage 3 or 4 CKD for all age groups. Additionally, although not statistically significant among the youngest age group, associations were present between hypertension and stage 3 or 4 CKD. However, for both hypertension and diagnosed diabetes, these associations were stronger among younger adults. Also associations between hypertension and diagnosed or undiagnosed diabetes mellitus with albuminuria were present across the adult lifespan but were stronger for younger adults. Also, a substantial proportion of adults = 70 years of age who were free of diabetes mellitus and hypertension had stage 3 or 4 CKD or albuminuria.

Recent studies have investigated differences in ESRD and mortality across age grouping among patients with stage 3 through 5 CKD not on dialysis.[5,6] In a study of over 2 million US veterans, the relative risk of mortality associated with lower eGFR was substantially higher for younger, compared to older, adults.[5] In a separate analysis of data for 209,622 patients with eGFR <60 ml/min/1.73 m^2 ^not on dialysis, the crude rates and multivariable-adjusted hazard ratios of ESRD within each eGFR strata was lower at older age.[13] Also, in a study from Southampton and South-West Hampshire, United Kingdom, the standardized mortality ratio of mortality was lower at older age for adults with persistent elevated serum creatinine (≥1.7 mg/dL for six months or longer).[14]

While several studies have investigated risk factors for CKD and albuminuria, few data are available on these associations by age group. In an analysis of the Cardiovascular Health Study, an observational study of community-dwelling adults ≥65 years of age at baseline, hypertension and cigarette smoking were associated with a decline in renal function among individuals who were free of diabetes mellitus.[15] In the Coronary Artery Risk Development in Young Adults study, a study of adults 18 to 28 years of age at baseline, glucose levels and change in systolic blood pressure were associated with elevated serum creatinine after 15 years of follow-up among both men and women. Also, among 2122 Bogalusa Heart Study participants with a mean age of 26.0 to 26.5 years, higher systolic and diastolic blood pressure was associated with an increased risk of albuminuria.[16] The current study extends these previous studies by demonstrating the importance of hypertension and diabetes mellitus as risk factors for stage 3 or 4 CKD and albuminuria across the full adult lifespan from age 20 through ≥80 years in a large nationally representative sample of US adults.

The weaker association of hypertension and diabetes mellitus with stage 3 or 4 CKD and albuminuria at older age and high prevalence of these conditions among older adults without hypertension or diabetes mellitus suggests that other factors may contribute to the high prevalence of kidney disease in older adults. For example, nephrotoxic agents that adults are exposed to over their life course may explain the higher rate of kidney disease as people age. Previous studies have reported environmental exposures including lead and cadmium are more common at older age and are associated with the development of kidney disease.[17,18] Also, older adults have higher rates of exposure to potentially nephrotoxic medications (e.g., acetaminophen) and medical tests (e.g., angiograms) that may increase their risk of kidney disease. [19-21] Additionally, local obstructive processes are more common in older adults and may play a role in the development of CKD and albuminuria.[22] Atherosclerosis is another possible factor contributing to the high burden of stage 3 or 4 CKD and albuminuria among older adults. Atherosclerosis is very common among older adults and can affect the renal vasculature resulting directly in renal damage.[23,24]. Also, the presence of more severe atherosclerosis may be a marker for developing heart failure resulting in decreased renal perfusion. A study by Shlipak and colleagues found, after multivariable adjustment, among adults ≥ 65 years of age without a history of clinical cardiovascular disease, an ankle-brachial index <0.9 was associated with a 1.61 times higher risk of a rapid decline in eGFR defined as ≤ -3 ml/min/1.73 m^2^/year.[25]. Additionally, participants in their study with a common carotid intima-medial thickness ≥ 1.19 and internal carotid intima-medial thickness ≥ 1.82 were 1.34 and 1.41 times more likely to have a rapid decline in eGFR, respectively. The current study did not have data on sub-clinical atherosclerosis, precluding investigation of increased levels of this risk factor on the development of stage 3 or 4 CKD and albuminuria.

As prevalence ratios depend on the baseline disease risk (i.e., the risk of disease in the unexposed group), the attenuation of prevalence ratios at older age may be a statistical anomaly resulting from the high prevalence of stage 3 or 4 CKD and albuminuria among older adults. However, the absolute difference in the prevalence of hypertension and diabetes mellitus for those with and without stage 3 or 4 CKD and albuminuria was also larger among younger versus older adults. The consistency of larger associations of hypertension and diabetes mellitus with stage 3 or 4 CKD and albuminuria among younger adults, (i.e., using absolute differences or prevalence ratios) highlights the importance of these risk factors among younger adults. Furthermore, the age-dependent association between hypertension and diabetes with CKD suggests that it may be a different physiologic condition when present in younger versus older adults. Future studies are needed to evaluate these differences by age.

The results of the current study demonstrate the importance of hypertension and diabetes mellitus on kidney disease in young adults. However, its important to recognize the low prevalence of stage 3 or 4 CKD among adults < 50 years of age with hypertension or diabetes mellitus. While a previous analysis showed population-wide screening for albuminuria was not cost-effective, there was a clear beneficial cost-effectiveness ratio for screening adults with hypertension or diabetes mellitus regardless of their age.[26] Also, a scientific advisory statement from the American Heart Association, developed in conjunction with the National Kidney Foundation, recommended measuring serum creatinine, to screen for stage 3 or 4 CKD, for all adults with hypertension or diabetes mellitus including those under 50 years of age.[27]

### Limitations and Strengths

The relatively small number of NHANES 1999–2004 study participants under 50 years of age with stage 3 or 4 CKD is a major limitation of the current analysis. As such, adequate statistical power was not available to detect small associations of risk factors with stage 3 or 4 CKD in this age group. An additional limitation is the cross-sectional study design of NHANES 1999–2004. Although we studied established risk factors for stage 3 or 4 CKD, caution should be taken when inferring causality based on the results of the current study. Also, as with any large-scale epidemiologic study, NHANES 1999–2004 relied on the MDRD study equation to estimate GFR and determine the presence of stage 3 or 4 CKD. Rule and colleagues reported that the MDRD underestimates GFR in healthy individuals. [28] The differential impact of the MDRD study equation on classifying CKD by age group warrants further study. Finally, we relied on fasting plasma glucose and not an oral glucose tolerance test to define the presence of diabetes mellitus. Therefore, we may have underestimated the prevalence of undiagnosed diabetes mellitus in our sample. However, it is unlikely that this occurred differentially by age grouping.

One of the major strengths of the current study was its large sample which permitted us to perform analyses for three separate age categories. In addition, NHANES 1999–2004 provides data that represents the non-institutionalized civilian US population that were collected following standardized protocols and data were available to analyze stage 3 or 4 CKD and albuminuria, separately. Few epidemiologic data are available on the presence of albuminuria among older adults. The consistency of the trends of lower prevalence ratios for both stage 3 or 4 CKD and albuminuria at older age is noteworthy.

## Conclusion

In conclusion, hypertension and diabetes mellitus are associated with stage 3 or 4 CKD and albuminuria across the full adult age range. Importantly, this association was statistically significantly stronger at younger age groupings. The low overall prevalence of stage 3 or 4 CKD among adults <50 years of age may mask the increased risk of CKD among individuals with hypertension or diabetes mellitus. Further research is needed to better define the differential impact of risk factors for the development of stage 3 or 4 CKD and albuminuria by age group. Additionally, studies aimed at the identification of novel risk factors for stage 3 or 4 CKD and albuminuria, especially among older adults, are warranted.

## Competing interests

The authors declare that they have no competing interests.

## Authors' contributions

TMI assisted with the conduct of the statistical analysis and conducted the initial drafting of, and revisions to, the manuscript. CSF participated in the conception of study hypothesis, review of statistical analyses, and provided critical review of the manuscript. DM provided a review of statistical analyses and a critical review of the manuscript. PM participated in the conception of the study hypothesis, conducted the statistical analysis, and provided a critical review of the manuscript. All authors read and approved the final manuscript.

## Pre-publication history

The pre-publication history for this paper can be accessed here:

http://www.biomedcentral.com/1471-2369/10/17/prepub

## Supplementary Material

Additional file 1 **Demographic characteristics and cardiovascular disease risk factors among NHANES 1999–2004 participants with and without stage 3–4 chronic kidney disease by age grouping**. The data provided represent the cardiovascular risk factors for young, middle aged, and older adults with and without chronic kidney disease. Click here for file

Additional file 2 **Prevalence ratios of stage 3–4 chronic kidney disease associated with selected risk factors by age group**. The data provided demonstrate a stronger association between risk factors and chronic kidney disease for younger, compared with older, adults. Click here for file

Additional file 3 **Demographic characteristics and cardiovascular disease risk factors among NHANES 1999–2004 with and without albuminuria by age grouping**. The data provided represent the cardiovascular risk factors for young, middle aged, and older adults with and without microalbuminuria. Click here for file

Additional file 4 **Prevalence ratios of albuminuria associated with selected risk factors by age group**. The data provided demonstrate a stronger association between risk factors and microalbuminuria for younger, compared with older, adults. Click here for file
