# Supplementary material for: Age-related associations of hypertension and diabetes mellitus with chronic kidney disease
Source: BMC Nephrol. 2009 Jun 30;10:17. doi: 10.1186/1471-2369-10-17 (PMC2714514; doi:10.1186/1471-2369-10-17)
Supplement: Additional file 1 — Demographic characteristics and cardiovascular disease risk factors among NHANES 1999–2004 participants with and without stage 3–4 chronic kidney disease by age grouping. The data provided represent the cardiovascular risk factors for young, middle aged, and older adults with and without chronic kidney disease. [file 1471-2369-10-17-S1.doc]

Table 1 – Demographic characteristics and cardiovascular disease risk factors among NHANES 1999-2004 participants with and without stage 3-4 chronic kidney disease by age grouping.

|  | Age 20 to 49 years | | | Age 50 to 69 years | | | Age ≥ 70 years | | |
| --- | --- | --- | --- | --- | --- | --- | --- | --- | --- |
|  | Stage 3 – 4 CKD  1.4%† | |  | Stage 3 – 4 CKD  9.9%† | |  | Stage 3 – 4 CKD  38.3%† | |  |
|  | No (N=6164) | Yes  (N=74) | p-value‡ | No  (N=3346) | Yes  (N=351) | p-value‡ | No  (N=1612) | Yes  (N=971) | p-value‡ |
| Mean age, years | 35.1 (0.2) | 41.9 (0.9) | <0.001 | 57.7 (0.1) | 60.5 (0.4) | <0.001 | 76.0 (0.1) | 78.8 (0.2) | <0.001 |
| Female gender, % | 48.8 | 54.3 | 0.670 | 50.2 | 62.8 | <0.001 | 57.7 | 62.5 | 0.119 |
| Black race, % | 11.7 | 12.5 | 0.540 | 9.3 | 6.8 | 0.087 | 7.5 | 5.9 | 0.034 |
| Mean systolic blood pressure, mmHg | 117.0 (0.3) | 122.8 (0.3) | 0.207 | 130.0 (0.5) | 129.8 (1.5) | 0.150 | 142.6 (0.9) | 145.1 (0.8) | 0.885 |
| Mean diastolic blood pressure, mmHg | 72.4 (0.2) | 77.1 (2.0) | 0.317 | 75.0 (0.3) | 73.3 (0.6) | 0.178 | 67.5 (0.5) | 65.5 (0.5) | 0.259 |
| On antihypertensive medications^, % | 46.8 | 66.6 | 0.140 | 67.2 | 85.0 | <0.001 | 60.0 | 75.3 | <0.001 |
| Mean body mass index, kg/m^2^ | 27.8 (0.1) | 29.0 (1.3) | 0.649 | 28.9 (0.2) | 30.4 (0.4) | 0.003 | 27.2 (0.2) | 27.6 (0.3) | 0.011 |
| Mean total cholesterol, mg/dL | 196.1 (0.7) | 205.6 (5.6) | 0.989 | 214.1 (0.8) | 212.0 (2.9) | 0.387 | 208.8 (1.3) | 206.4 (1.6) | 0.313 |
| On cholesterol lowering medications^^, % | 19.1 | 40.0 | 0.053 | 48.1 | 60.7 | 0.024 | 59.7 | 62.0 | 0.035 |
| Cigarette smokers, % | 24.9 | 16.2* | 0.178 | 18.5 | 16.8 | 0.914 | 5.3 | 6.9 | 0.990 |
| Obese, % | 28.5 | 28.7 | 0.642 | 34.8 | 43.9 | 0.008 | 23.3 | 25.5 | 0.004 |
| Hypertension, % | 13.2 | 35.4 | 0.067 | 45.3 | 62.1 | 0.008 | 67.9 | 77.8 | <0.001 |
| High cholesterol, % | 15.6 | 30.6 | 0.134 | 38.4 | 47.8 | 0.044 | 42.3 | 43.3 | 0.046 |
| Diabetes mellitus |  |  |  |  |  |  |  |  |  |
| Diagnosed diabetes, % | 2.1 | 11.2* | 0.006 | 9.6 | 18.1 | 0.004 | 10.3 | 15.7 | 0.002 |
| Undiagnosed diabetes, % | 1.9 | 6.9* | 0.315 | 6.6 | 9.5 | 0.500 | 7.7 | 8.7 | 0.620 |
| Prevalent Cardiovascular disease | 1.3 | 3.9* | 0.131 | 7.8 | 17.9 | <0.001 | 16.1 | 25.5 | <0.001 |

†Weighted stage 3 or 4 CKD prevalence calculated by applying NHANES 1999-2004 sampling weights

Abbreviations: CKD – chronic kidney disease

Numbers in table are mean (standard error) or percentage

‡ p-values are age adjusted

^ Among participants with hypertension

^^ Among participants with high cholesterol

* Estimate may not be reliable due to the small number of individuals (n<30) in this sub-group.
