# Supplementary material for: Age-related associations of hypertension and diabetes mellitus with chronic kidney disease
Source: BMC Nephrol. 2009 Jun 30;10:17. doi: 10.1186/1471-2369-10-17 (PMC2714514; doi:10.1186/1471-2369-10-17)
Supplement: Additional file 2 — Prevalence ratios of stage 3–4 chronic kidney disease associated with selected risk factors by age group. The data provided demonstrate a stronger association between risk factors and chronic kidney disease for younger, compared with older, adults. [file 1471-2369-10-17-S2.doc]

Table 2 - Prevalence ratios of stage 3 -4 chronic kidney disease associated with selected risk factors by age group.

|  | Age 20 to 49 years | Age 50 – 69 years | Age ≥ 70 years | P-trend |
| --- | --- | --- | --- | --- |
| Black race† | 0.95 (0.58 – 1.55) | 0.65 (0.46 – 0.92) | 0.79 (0.69 – 0.91) | 0.264 |
| Female gender | 1.22 (0.71 – 2.10) | 1.56 (1.24 – 1.96) | 1.06 (0.95 – 1.18) | 0.332 |
| Cigarette smokers | 0.62 (0.28 – 1.38)* | 1.09 (0.76 – 1.57) | 1.07 (0.81 – 1.40) | 0.255 |
| Obese | 0.66 (0.35 – 1.23) | 1.19 (0.95 – 1.50) | 1.18 (1.04 – 1.34) | 0.125 |
| Hypertension | 1.94 (0.86 – 4.35) | 1.51 (1.09 – 2.07) | 1.31 (1.15 – 1.49) | 0.038 |
| High cholesterol | 1.42 (0.82 – 2.48) | 1.18 (0.92 – 1.53) | 1.07 (0.97 -1.18) | 0.087 |
| Diabetes mellitus |  |  |  |  |
| Diagnosed diabetes | 3.01 (1.35 – 6.74)* | 1.61 (1.15 – 2.25) | 1.40 (1.15 – 1.69) | 0.067 |
| Undiagnosed diabetes | 2.67 (0.53 – 13.4)* | 1.35 (0.69 – 2.63) | 1.08 (0.78 – 1.51) | 0.369 |
| Prevalent cardiovascular disease | 1.61 (0.78 – 3.29)* | 1.93 (1.37 – 2.72) | 1.27 (1.14 – 1.42) | 0.018 |

† Reference group includes individuals other than blacks (i.e. whites, Mexican-Americans and individuals of other race-ethnicities)

Numbers in table represent prevalence ratio (95% confidence interval)

Adjusted for age, race, sex, hypertension and self-reported diabetes (except hypertension which is adjusted for age, race, sex, and diabetes and diabetes which is adjusted for age, race, sex and hypertension)

* Estimate may not be reliable due to the small number of individuals (n<30) in this sub-group.
