# Supplementary material for: Age-related associations of hypertension and diabetes mellitus with chronic kidney disease
Source: BMC Nephrol. 2009 Jun 30;10:17. doi: 10.1186/1471-2369-10-17 (PMC2714514; doi:10.1186/1471-2369-10-17)
Supplement: Additional file 4 — Prevalence ratios of albuminuria associated with selected risk factors by age group. The data provided demonstrate a stronger association between risk factors and microalbuminuria for younger, compared with older, adults. [file 1471-2369-10-17-S4.doc]

Table 4 - Prevalence ratios of albuminuria associated with selected risk factors by age group.

|  | Age 20 to 49 years | Age 50 – 69 years | Age ≥ 70 years | P-trend |
| --- | --- | --- | --- | --- |
| Black race† | 1.28 (1.07 – 1.54) | 1.38 (1.10 – 1.73) | 1.13 (0.96 – 1.32) | 0.192 |
| Female gender | 1.31 (1.08 – 1.58) | 0.87 (0.72 – 1.04) | 0.77 (0.65 – 0.90) | 0.001 |
| Cigarette smokers | 1.34 (1.02 – 1.76) | 1.32 (1.05 – 1.66) | 1.60 (1.26 – 2.03) | 0.018 |
| Obese | 1.35 (1.02 – 1.79) | 1.22 (0.96 – 1.55) | 1.17 (1.01 – 1.36) | 0.609 |
| Hypertension | 2.70 (1.96 – 3.73) | 1.67 (1.31 – 2.12) | 1.54 (1.19 – 2.00) | 0.019 |
| High cholesterol | 1.14 (0.81 – 1.61) | 0.95 (0.79 – 1.14) | 0.82 (0.69 – 0.96) | 0.060 |
| Diabetes mellitus |  |  |  |  |
| Diagnosed diabetes | 3.53 (2.32 – 5.36) | 3.41 (2.78 – 4.19) | 1.70 (1.37 – 2.10) | <0.001 |
| Undiagnosed diabetes | 5.38 (2.86 – 10.2) | 3.17 (2.00 – 5.03) | 1.57 (1.02 – 2.41) | 0.003 |
| Prevalent cardiovascular disease | 1.09 (0.47 – 2.52)* | 1.33 (1.03 – 1.72) | 1.09 (0.88 – 1.36) | 0.729 |

† Reference group includes individuals other than blacks (i.e. whites, Mexican-Americans and individuals of other race-ethnicities)

Numbers in table represent prevalence ratio (95% confidence interval)

Adjusted for age, race, sex, hypertension and self-reported diabetes (except hypertension which is adjusted for age, race, sex, and diabetes and diabetes which is adjusted for age, race, sex and hypertension)

* Estimate may not be reliable due to the small number of individuals (n<30) in this sub-group.
